# Antimicrobial Combinations against Pan-Resistant *Acinetobacter baumannii* Isolates with Different Resistance Mechanisms

**DOI:** 10.1371/journal.pone.0151270

**Published:** 2016-03-21

**Authors:** Gleice Cristina Leite, Maura Salaroli Oliveira, Lauro Vieira Perdigão-Neto, Cristiana Kamia Dias Rocha, Thais Guimarães, Camila Rizek, Anna Sara Levin, Silvia Figueiredo Costa

**Affiliations:** 1 Department of Infectious Diseases, University of São Paulo, São Paulo, Brazil; 2 Laboratory of Medical Investigation 54 (LIM-54), São Paulo, Brazil; 3 Hospital Das Clínicas FMUSP, São Paulo, Brazil; 4 Department of Infection Control, Hospital das Clínicas, University of São Paulo, São Paulo, Brazil; University Medical Center Utrecht, NETHERLANDS

## Abstract

The study investigated the effect of antibiotic combinations against 20 clinical isolates of *A*. *baumannii* (seven colistin-resistant and 13 colistin-susceptible) with different resistance mechanisms. Clinical data, treatment, and patient mortality were evaluated. The following methods were used: MIC, PCRs, and outer membrane protein (OMP) analysis. Synergy was investigated using the checkerboard and time-kill methods. Clonality was evaluated by PFGE. Based on clonality, the whole genome sequence of six *A*. *baumannii* isolates was analyzed. All isolates were resistant to meropenem, rifampicin, and fosfomycin. OXA-23 and OXA-143 were the most frequent carbapenemases found. Four isolates showed loss of a 43kDa OMP_._ The colistin-susceptible isolates belonged to different clones and showed the highest synergistic effect with fosfomycin-amikacin. Among colistin-resistant isolates, the highest synergistic effect was observed with the combinations of colistin-rifampicin followed by colistin-vancomycin. All colistin-resistant isolates harbored *bla*_OXA-23-like_ and belonged to CC113. Clinical and demographic data were available for 18 of 20 patients. Fourteen received treatment and eight patients died during treatment. The most frequent site of infection was the blood in 13 of 14 patients. Seven patients received vancomycin plus an active drug against *A*. *baumannii*; however, mortality did not differ in this group. The synergistic effect was similar for colistin-susceptible isolates of distinct clonal origin presenting with the same resistance mechanism. Overall mortality and death during treatment was high, and despite the high synergism *in vitro* with vancomycin, death did not differ comparing the use or not of vancomycin plus an active drug against *A*. *baumannii*.

## Introduction

Infections caused by multidrug-resistant *Acinetobacter baumannii* have emerged as a serious problem throughout the world [1]. Old antibiotics, such as fosfomycin and polymyxins, are now considered potential treatment alternatives to overcome the lack of new antibiotics [2–4]. Studies have demonstrated that fosfomycin is a promising drug, particularly in combination with other antimicrobials for the treatment of infections due to multidrug-resistant (MDR) Gram-negative bacilli. However, there is concern about its use against *A*. *baumannii*, due to intrinsic resistance to fosfomycin [5–6]. On the other hand, although polymyxins B and E (colistin) are generally active against multidrug-resistant *A*. *baumannii* (MDRAB) [3] and have been used to treat infections, colistin resistance among *A*. *baumannii* has been reported and has clearly increased in the last years [4]. In this scenario, treatment with combination therapy, using two or more antibacterial drugs, appears to be the only remaining option [7].

Two of the most frequent *in vitro* methods used to evaluate interactions between drugs are the checkerboard technique and time-kill kinetics. The checkerboard method only evaluates the inhibitory activity, not bactericidal activity. Additionally, it shows different results when different methods of interpretation are used [8]. Thus, its results may require confirmation using a more dynamic interaction method such as time-kill kinetic studies. To date, few studies have evaluated the antimicrobial combinations against pan-resistant *A*. *baumannii* isolates using both methods, and correlations between *in vitro* and *in vivo* results are often controversial. There is also some concern as to whether the synergistic effect of antibiotics is related to the resistance mechanism or to the clonality of isolates, or both [9]. Thus, data on the synergistic effect of antibiotic combinations and their efficacy in the treatment of infections caused by *Acinetobacter* are needed.

The objective of this study was to evaluate the *in vitro* activity of antibiotic combinations against twenty MDRAB, including pan-resistant isolates with different resistance mechanisms and clonal origins. In addition, clinical and demographic data of patients submitted to different treatments against these infections were evaluated.

## Methods

### Bacterial Isolates

Twenty *A*. *baumannii* isolates were obtained from the bacterial collection of the Laboratory of Bacteriology (LIM-54) of the Department of Infectious Diseases of the School of Medicine, University of São Paulo. Thirteen isolates were colistin-susceptible (at 0.5 mg/L to 2 mg/L) and seven were colistin-resistant (at 8 mg/L to 64 mg/L). Isolates had been stored at -80°C and were subcultured on 5% sheep blood agar before being tested.

### Susceptibility testing

Minimal Inhibitory Concentrations **(**MICs) of colistin (USP-Reference Standard, Rockville, MD, USA), rifampicin, imipenem, gentamycin, amikacin, tigecycline, fosfomycin, vancomycin (Sigma-Aldrich, St Louis, MO, USA), and meropenem (Astra Zeneca, Cotia, SP, Brazil) were determined using the broth microdilution method in duplicate according to the Clinical and Laboratory Standards Institute (CLSI) protocol [10]. Breakpoints for fosfomycin were used according to EUCAST criteria for *Enterobacteriaceae* [11]. These antibiotics were selected based on local therapeutic protocols used at the Hospital das Clínicas of the University of São Paulo. *Pseudomonas aeruginosa* ATCC 27853 and *Escherichia coli* ATCC 25922 were used as controls for susceptibility testing. Pan-resistance was defined as resistance to all antimicrobials tested, and multidrug-resistance was defined as resistance to at least three antimicrobials tested.

### Polymerase chain reaction (PCR)

The PCR techniques for carbapenemase genes (*bla*_OXA-23_-_*like*_; *bla*_OXA-51_-_*like*_; *bla*_OXA-58-like_; *bla*_OXA-24-like_; *bla*_OXA-143-like_*; bla*_IMP_; *bla*_SPM_; *bla*_VIM_; *bla*_SIM_; *bla*_NDM_), and the genes that encode the omp33-36 porin and *car*O were performed in duplicate; other genes described in *A*. *baumannii* were verified by whole genome sequence. DNA were extracted using Illustra bacteria genomicPrep Mini Spin Kit (GE Healthcare Bio-Sciences Corp, USA). For each reaction, a positive internal control was used. The primers used in the study are listed on Table 1.

**Table 1:** DNA sequences of oligonucleotides used in polymerase chain reactions to detect carbapenemase and outer-membrane proteins of *A*. *baumannii* isolates.

Oligonucleotides	Sequence (5’-3’)	Size (bp)	Reference
***bla***_**OXA51**_**—F**	TAATGCTTTGATCGGCCTTG	353	[22]
***bla***_**OXA51**_**—R**	TGGATTGCACTTCATCTTGG		
***bla***_**OXA23**_**—F**	GATCGGATTGGAGAACCAGA	501	[22]
***bla***_**OXA23**_**—R**	ATTTCTGACCGCATTTCCAT		
***bla***_**OXA24**_**—F**	GGTTAGTTGGCCCCCTTAAA	246	[22]
***bla***_**OXA24**_**—R**	AGTTGAGCGAAAAGGGGATT		
***bla***_**OXA58**_**—F**	AAGTATTGGGGCTTGTGCTG	599	[22]
***bla***_**OXA58**_**—R**	CCCCTCTGCGCTCTACATAC		
***bla***_**OXA143**_**—F**	AGTTAACTTTCAATAATTG	604	[23]
***bla***_**OXA143**_**—R**	TTGGAAAATTATATAATCCC		
***bla***_**SPM**_**—F**	CCTTTTCCGCGACCTTGATC	798	[24]
**bla**_**SPM**_**—R**	ATGCGCTTCATTCACGCAC		
***bla***_**SIM**_**—F**	GTACAAGGGATTCGGCATCG	569	[24]
***bla***_**SIM**_**—R**	GTACAAGGGATTCGGCATCG		
***bla***_**IMP**_**—F**	TTGGAAAATTATATAATCCC	188	[24]
***bla***_**IMP**_**—R**	CCAAACCACTAGGTTATC		
***bla***_**VIM**_**—F**	TTTGGTCGCATATCGCAAAG	382	[24]
***bla***_**VIM**_**—R**	CCATTCAGCCCAGATCGGCAT		
***bla***_**NDM**_**—F**	GGCGGAATGGCTCATCACGA	375	[25]
***bla***_**NDM**_**—R**	CGCAACACAGCCTGACTTTC		
**omp33-36—F**	CATCGATGGCACTAACATGG	175	[25]
**omp33-36—R**	AGTGTGACCACCCCAAACAT		
***car*O—F**	GGTTACGGTGGTGCTTTGTT	118	[25]
***car*O—R**	GGTGCGAAACCAAAACCTAA		

### Analysis of outer membrane proteins

Bacterial cells were obtained from overnight Brain Heart Infusion (BHI) cultures of *A*. *baumannii*. Extraction of outer membrane proteins as well as the analysis of PAGE was performed in duplicate, using a previously described method [12]. *Acinetobacter baumannii* ATCC 19606 was used as a control.

### Pulsed-field gel electrophoresis (PFGE)

Clonality of *A*. *baumannii* isolates was evaluated using PFGE with *Apa*I endonuclease (Applied Biosystems, Foster City, California, USA) and chromosomal DNA Ultrapure Agarose (Invitrogen^™^, Life Technologies) [13]. Restriction fragments were obtained by separation using a CHEF DR^®^III system (Bio-Rad, Hercules, California, USA). Patterns were interpreted according to Bionumerics version 7.1 (Applied-Maths, Sint-Martens-Latem, Belgium). Results from our previous study [12] allowed us to select distinct clones of colistin-susceptible isolates, and to examine the possible different synergistic effects. *Acinetobacter baumannii* ATCC 19606 were used as controls.

### Whole genome sequencing

Genomic DNA was extracted using the Illustra bacteria genomicPrep Mini Spin Kit (GE Healthcare Bio-Sciences Corp, USA). An Ion Torrent adapter-ligated library was made following the manufacturer's Ion AmpliSeq Library Kit (Life Technologies). The whole-genome sequence was determined using Ion Torrent Personal Genome Technology MachineTM (PGM) system with a 318 chip (Life Technologies, Foster City, CA). Raw sequencing reads were quality-controlled using Trimmomatic [14]. Draft genomes were *de novo* assembled using MIRA [15]. Genome annotation was performed using Prokka [16], and rRNA was identified using RNAmmer [17]. The ResFinder 2.0 server (http://cge.cbs.dtu.dk/services/ResFinder/) was used to identify antibiotic resistance genes. Comparative analysis were made using BLAST (www.ncbi.nlm.nih.gov/blast/) by alignment and by MAFFT (http://mafft.cbrc.jp/alignment/server/) to verify amino acid replacement [18].

### MLST (Multilocus sequence typing)

MLST was determined by *in silico* analysis of the draft genomes using the *A*. *baumannii* MLST database (http://pubmlst.org/abaumannii), and Clonal Complexes were analyzed by eBURST software (http://eburst.mlst.net/) [19].

### Checkerboard microdilution

The MDR *A*. *baumannii* isolates were exposed to combinations of two drugs, and checkerboard microdilution testing was performed in duplicate and evaluated after 20–24 hours of incubation at 35°C. Growth and sterility controls were tested in all plates. Colistin, imipenem, fosfomycin, and tigecycline were combined with amikacin, gentamycin, rifampicin, vancomycin, and meropenem at the respective minimum inhibitory concentrations determined by microdilution. The antimicrobial agents were diluted from the stock solution, and left at concentrations 4 times higher than the final concentration in plate, and then a serial dilution was performed. FICI and 2-well interpretation methods were used as described by Eliopoulos et al. [20]. FICIs were calculated as [(MIC of drug A in combination)/(MIC of drug A alone)] + [(MIC of drug B in combination)/(MIC of drug B alone)]. Synergy was defined as a FICI of ≤0.5, indifference as a FICI of >0.5<4, and antagonism as a FICI of ≥4. The second method used was the 2-well method, with synergy defined as the absence of growth in wells containing 0.25 x MIC of both drugs and 2 x MIC of both drugs [8, 20].

### Time-Kill assay

Time-kill assays were performed in duplicate for all isolates at concentrations based on the MIC determined from the checkerboard testing of isolates as follows: drugs alone and combined at 1 x MIC and 0.5 x MIC. For isolates with indifferent effect and for colistin-resistant isolates, they were performed combined at 0.125 x MIC, 0.25 x MIC, 2 x MIC, and 4 x MIC. Time-kill analysis was performed based on a previously published method [9]. The tests were repeated for the third time with the isolates that had grown at 24 hours. Synergism was interpreted as a ≥ 2 log_10_ decrease in colony count with the antimicrobial combination when compared to the most active single agent. The drug combination was considered to be antagonistic if there was a ≥2 log_10_ increase in counts, and the combination was considered to be indifferent if there was a < 2 log_10_ increase or decrease in colony count with the combination when compared with the most active drug alone [9].

### Clinical and demographic data

The following clinical and demographic data from the medical records of patients hospitalized at Hospital das Clínicas of the University of São Paulo, Brazil, were registered: age, gender, underlying diseases, site of infection, length of stay in the Intensive Care Unit, APACHE II score, and treatment of *A*. *baumannii* infection (with at least 48 hours of use of a specific antibiotic with activity against *A*. *baumannii*). Definitions for the infections were those used by the Centers for Disease Control and Prevention [21].

The *in vitro* antimicrobial combinations of vancomycin plus colistin, and vancomycin plus imipenem or meropenem against *A*. *baumannii* were compared using demographic data. We also evaluated patient mortality during treatment (if the patient was receiving specific treatment for *A*. *baumannii*) and during hospitalization as outcomes. An Epi Info^™^ database was built, and results were expressed as means (standard deviation) or median (interquartile range), depending on normality. Statistical analysis was not done due to the limited number of patients. All data were analyzed anonymously and confidentially, with approval by the Research Ethics Committee of the FM-USP (School of Medicine, University of São Paulo).

## Results

All 20 isolates of *A*. *baumannii* were resistant to meropenem, rifampicin, and fosfomycin. All isolates of *A*. *baumannii* harbored *bla*_OXA-51-like_, 10 carried the *bla*_OXA-23-like_ gene, seven carried *bla*_OXA-143-like_, and three carried the *bla*_IMP_ gene. The *bla*_SPM_, *bla*_VIM_ and *bla*_SIM_ genes were not identified by PCR. The *bla*_SPM_, *bla*_VIM_ and *bla*_SIM_ genes were not identified. Sixteen isolates carried the opm33-36 gene and all isolates carried the *car*O gene. For all the isolates harboring *bla*_OXA-23-like,_ the combination of colistin-vancomycin was synergistic. Synergism was present in 80% with the colistin-imipenem combination, and in 80% with fosfomycin-amikacin. For all the isolates that harbored *bla*_OXA-143-like_, the fosfomycin-amikacin combination was synergistic, and 85.7% presented synergism for the tigecycline-amikacin combination.

The absence or decrease of outer-membrane proteins of 43 kDa, 33–36 kDa, and 29 kDa of the 20 isolates were compared with *A*. *baumannii* ATCC 19606. In four colistin-susceptible isolates, a total absence of the protein of 43kDa was observed. These four isolates presented with high MICs for carbapenem and harbored *bla*_OXA-51-like_, *bla*_OXA-143-like_, and *bla*_IMP_. Table 2 shows the MIC of the antibiotics tested and the resistance mechanisms of *A*. *baumannii* isolates determined by PCR and OMP evaluation. The interpretation criteria for antimicrobial susceptibility established by CLSI and EUCAST are shown on S1 Table.

**Table 2:** Minimum inhibitory concentration of antibiotics and resistance mechanisms of 20 *A*. *baumannii* isolates.

Isolates	MIC (mg/L)								PCR (OXA/Metallo)				OMP (kDa)		
	Col	Imi	Tig	Gen	Ami	Mer	^a^Rif	^b^Fos	51	23	143	IMP	29	33–36	43
1	2	256	1	32	128	64	4	128	P	A	A	A	+++	+++	+++
2	2	32	0.5	32	128	128	4	128	P	A	A	A	+	+++	+++
3	1	1	1	2	128	128	4	128	P	A	A	A	+++	+++	+++
4	2	32	0.5	64	128	128	4	128	P	A	P	A	+	+++	+
5	2	16	4	128	64	64	4	128	P	A	A	A	+++	++++	+++
6	1	128	1	32	128	128	4	128	P	A	P	P	+	++	A
9	0.5	128	1	16	128	64	8	256	P	A	A	P	+++	+++	+++
11	0.5	64	0.5	2	128	16	4	128	P	P	P	A	+	+++	A
13	0.5	64	0.25	64	128	32	4	128	P	A	P	A	A	+++	A
14	0.5	64	0.5	64	128	64	4	128	P	A	P	P	+	++++	+
15	0.5	128	2	64	128	64	2	128	P	A	A	A	+	+	+
18	1	16	0.5	2	256	16	4	128	P	P	P	A	+++	+++	+++
20	0.5	128	0.5	64	256	128	4	128	P	P	P	A	+	+++	A
22	16	32	2	4	256	32	4	64	P	P	A	A	++	+++	+++
23	16	32	2	4	256	32	2	128	P	P	A	A	++	+++	+++
24	32	32	2	4	512	32	2	64	P	P	A	A	++	+++	+++
25	64	64	16	16	512	32	4	32	P	P	A	A	++	+++	+++
26	32	32	2	4	128	32	2	64	P	P	A	A	++	+++	+++
27	32	64	0.5	2	2	16	4	>256	P	P	A	A	++	+++	+++
28	8	32	2	8	256	16	4	64	P	P	A	A	++	+++	+++

Abbreviations: Col: colistin; Imi: imipenem; Tig: tigecycline; Gen: gentamycin; Ami: amikacin; Mer: meropenem; Rif: rifampicin; Fos: fosfomycin; P: presence; OMP: Outer Membrane Protein; MIC: Minimum Inhibitory Concentration;

^a^Rif: rifampicin, criteria established by EUCAST (breakpoint ≤0.006 mg/L),

^b^Fos: fosfomycin (breakpoint ≤32 mg/L).

The colistin-susceptible *A*. *baumannii* isolates showed eight distinct profiles and the colistin-resistant isolates showed three distinct profiles by PFGE.

There were two distinct ST profiles among colistin-susceptible isolates; two colistin-susceptible isolates were assigned to ST236 (CC103), and one to ST406 (singleton). The three colistin-resistant isolates were assigned to ST233, a member of the CC113. These ST are unrelated to international clones I, II, and III (Table 3).

**Table 3:** Determination of minimum inhibitory concentration by microdilution, resistance genes, and molecular profile of six *A*. *baumannii* isolates.

Isolates			*MIC (μg/mL)								MOLECULAR PROFILE			
	Unit/Year	Isolation Site	Col	Imi	Mer	Tig	Ami	Gen	Rif	Fos	Genes	Cluster	ST	CC
03	Clinical ICU**2003	Blood	1	1	128	1	128	2	4	128	OXA-51, ampC, adeABC, adeIJK, aph(3´)-Vla	C	236	103
06	Nursery I2002	Blood	1	128	128	1	128	32	4	128	OXA-51, OXA-182, ampC, IMP, adeABC, adeIJK, aph(3´)-Vla, aadA1	C	236	103
18	Nursery II 2004	Blood	1	16	16	0.5	256	2	4	128	OXA-51, OXA-23, OXA-72, ampC, adeABC, adeIJK, aph(3´)-Vla	D	407	St
23	Surgical ICU2012	Rectal Swab	16	32	32	2	256	4	2	128	OXA-51, OXA-23, OXA-27, ampC, TEM, adeABC, adeIJK, aph(3´)-Vla, aadA1, strA, strB	A	233	133
25	Neurological ICU2011	Blood	64	64	32	16	512	16	4	32	OXA-51, OXA-23, OXA-88, ampC, TEM, adeABC, adeIJK, aph(3´)-Vla, aadA1, strA, strB	A2	233	133
28	Surgical ICU2011	Blood	8	32	16	2	256	8	4	64	OXA-51, OXA-23, ampC, TEM, adeABC, adeIJK, aph(3´)-Vla, aadA1, strB	B	233	133

*According to CLSI, 2013 and EUCAST, 2013. St: singletons,

**ICU: intensive care unit

Based on the FICI method, the synergistic effect was observed only in one isolate for the colistin-vancomycin combination, and in all colistin resistant isolates (n = 7) for the colistin-rifampicin combination.

All the isolates harboring *bla*_OXA-23-like_ displayed a synergistic effect of the colistin-vancomycin combination. Four isolates of *A*. *baumannii*, in which the protein of 43 kDa was absent, had a high MIC for imipenem, and the combinations with carbapenems were indifferent. Also, all isolates presented synergism for the fosfomycin-amikacin and tigecycline-amikacin combinations.

Table 4 shows the effect of antimicrobial combinations by the checkerboard (FICI and 2-well) and time-kill methods of the combinations tested that showed the largest number for synergic effect. In these antimicrobial combinations, synergism was observed in 100% of isolates by the time-kill assay. Antagonism was not noted.

**Table 4:** Results of the three different methods (FICI, 2-well, and time-kill assay) used to evaluate *in vitro* synergism of antibiotic combinations against 20 *A*. *baumannii* isolates.

Isolates	Main results																	
	Col+Imi			Col+Van			Col+Mer			Col+Rif			Fos+Gen			Fos+Ami		
	^a^FICI	2well	^b^TK	^a^FICI	2well	^b^TK	^a^FICI	2well	^b^TK	^a^FICI	2well	^b^TK	^a^FICI	2well	^b^TK	^a^FICI	2well	^b^TK
**1**	1.5/I	I	2/2h	0.75/I	S	3/2h	1.25/I	S	2/2h	0.31/S	S	6/2h	1/I	S	3/2h	1.5/I	S	2/2h
**2**	2/I	I	2/2h	1/I	S	4/2h	2/I	I	2/2h	0.5/S	S	3/2h	2/I	S	3/2h	1.25/I	S	2/2h
**3**	3/I	I	6/4h	1.5/I	I	3/2h	1/I	I	2/2h	0.5/S	S	6/2h	2/I	S	6/2h	1.25/I	S	2/2h
**4**	1.5/I	I	2/2h	0.75/I	I	3/2h	2/I	S	2/2h	1.5/I	S	3/2h	2/I	S	4/2h	1.25/I	S	2/2h
**5**	2/I	I	6/2h	0.75/I	S	3/2h	2/I	I	2/2h	1/I	S	2/2h	1.5/I	I	3/2h	1.5/I	S	3/2h
**6**	1.5/I	I	2/2h	1.5/I	S	4/2h	1.25/I	S	2/2h	1/I	S	4/2h	1.5/I	S	4/2h	1.25/I	S	2/2h
**9**	1.5/I	S	2/2h	1.5/I	S	4/2h	2/I	I	2/2h	1/I	I	2/2h	0.562/I	S	4/2h	1.25/I	S	2/2h
**11**	2/I	I	2/2h	1/I	S	4/2h	2/I	I	2/2h	0.75/I	I	2/2h	2/I	I	3/2h	1.25/I	S	2/2h
**13**	1.5/I	I	6/2h	2/I	I	4/2h	2/I	I	2/2h	1/I	I	2/2h	1.5/I	S	4/2h	1.5/I	S	2/2h
**14**	2/I	I	2/2h	1.5/I	I	3/2h	2/I	I	2/2h	1/I	I	2/2h	1.25/I	S	4/2h	2/I	S	2/2h
**15**	2/I	I	2/2h	1.5/I	S	4/2h	2/I	I	2/2h	1.5/I	I	2/2h	1.5/I	S	2/2h	1.5/I	S	2/2h
**18**	1.5/I	S	2/2h	2/I	S	4/2h	2/I	I	2/2h	1.25/I	I	2/2h	2/I	I	4/2h	1.5/I	S	2/2h
**20**	2/I	I	2/2h	0.75/I	S	4/2h	2/I	I	2/2h	1.5/I	I	2/2h	1.5/I	S	3/2h	1/I	S	2/2h
**22**	0.75/I	S	3/2h	0.625/I	S	4/2h	1.25/I	S	6/2h	0.135/S	S	6/2h	2/I	I	4/2h	1.25/I	S	2/2h
**23**	0.625/I	S	2/2h	0.507/I	S	4/2h	1.125/I	S	2/2h	0.155/S	S	5/2h	2/I	I	4/4h	1.5/I	S	2/2h
**24**	1.5/I	S	3/2h	0.625/I	S	6/2h	1.25/I	S	2/2h	0.132/S	S	6/2h	2/I	I	4/4h	1.5/I	S	2/2h
**25**	2.25/I	S	2/2h	1.125/I	S	6/2h	1/I	S	6/2h	0.135/S	S	3/2h	0.75/I	S	3/2h	1.5/I	S	2/2h
**26**	0.75/I	S	2/2h	1.062/I	S	5/2h	1/I	S	5/2h	0.135/S	S	6/2h	2/I	I	4/2h	1.5/I	S	2/2h
**27**	1.25/I	S	4/2h	1.25/I	S	4/2h	1/I	S	6/2h	0.135/S	S	6/2h	1.5/I	S	4/2h	2/I	I	2/2h
**28**	0.75/I	S	2/2h	0.122/S	S	4/2h	1.062/I	S	2/2h	0.185/S	S	3/4h	1.03/I	S	3/2h	2/I	I	2/2h

Abbreviations: Col: colistin; Imi: imipenem; Mer: meropenem; Gen: gentamycin; Ami: amikacin; Rif: rifampicin; Fos: fosfomycin; I: indifferent; S: synergism; h: hour.

^a^FICI (Fractional Inhibitory Concentration Index): value/effect;

^b^TK (Time-Kill): log decrease/bactericidal effect hour.

The percentage of synergistic effect using FICI and 2-well for all antimicrobial combinations against *A*. *baumannii* isolates is shown on Table 5.

**Table 5:** *In vitro* synergistic effect, according to two different methods (FICI and 2-well) of antimicrobial combinations against 20 isolates of multi-drug resistant *Acinetobacter baumannii*.

Antibiotic	Antibiotic tested in combination	Colistin-Susceptible (n = 13)		Colistin-Resistant (n = 7)	
		*Synergism (%)		*Synergism (%)	
		**FICI	2-Well	**FICI	2-Well
**Colistin**					
	Imipenem	0	15	0	**100**
	Tigecycline	0	15	0	0
	Gentamycin	0	23	0	0
	Amikacin	0	15	0	14
	Meropenem	0	23	0	**100**
	Vancomycin	0	**69**	14	**100**
	Rifampicin	23	**77**	**100**	**100**
	Fosfomycin	0	54	0	0
**Imipenem**					
	Tigecycline	0	31	0	43
	Gentamycin	0	54	0	29
	Amikacin	0	46	0	14
	Vancomycin	0	0	0	0
	Rifampicin	0	39	0	0
	Fosfomycin	0	23	0	0
**Tigecycline**					
	Gentamycin	0	8	0	29
	Amikacin	0	46	0	14
	Meropenem	0	8	0	29
	Vancomycin	0	8	0	0
	Rifampicin	0	0	0	0
	Fosfomycin	0	0	0	0
**Fosfomycin**					
	Gentamycin	0	**77**	0	43
	Amikacin	0	**100**	0	**71**
	Meropenem	0	23	0	29
	Vancomycin	0	23	0	14
	Rifampicin	0	39	0	0

*Synergism is a positive interaction; the combined effect of the drugs being examined is significantly greater than the expected result. The isolates that showed no synergistic effect showed indifference; antagonism was not observed for any combination against any isolate tested.

**FICI—Fractional Inhibitory Concentration Index.

**Fig 1:**
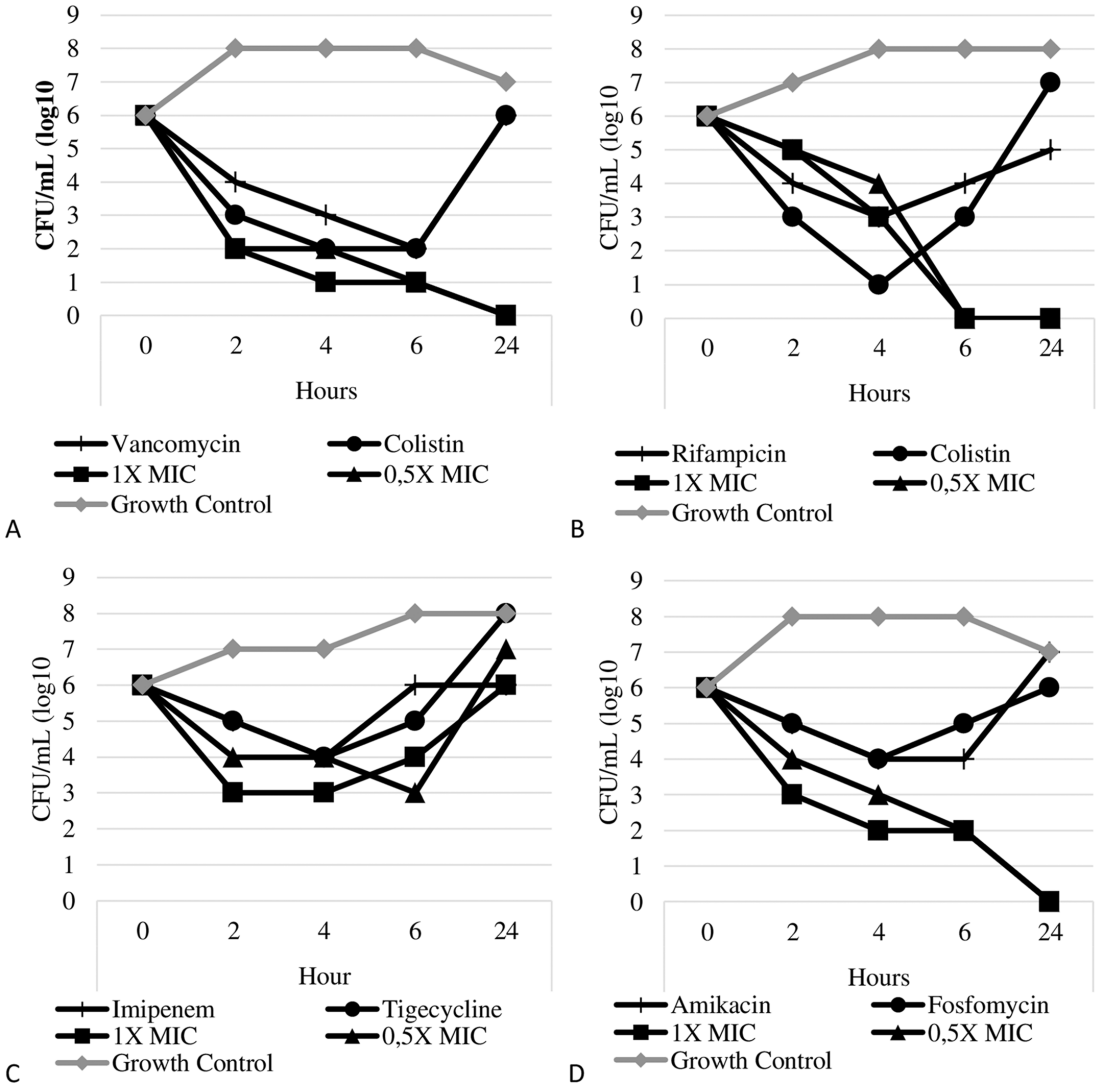
Time-kill curve of isolates using drugs alone and in combination at 1 x MIC and 0.5 x MIC, respectively, against an *A*. *baumannii* isolate. (A) Isolate 23 –Colistin (16 and 8 mg/L)/Vancomycin (64 and 32mg/L), (B) Isolate 28—Colistin (8 and 4 mg/L)/Rifampicin (4 and 2mg/L), (C) Isolate 1—Imipenem (256 and 128mg/L)/Tigecycline (1 and 0.5mg/L), (D) Isolate 5—Fosfomycin (128 and 64mg/L)/Amikacin (64 and 32mg/L).

The time-kill results confirmed a synergistic effect for all isolates with a synergistic effect by checkerboard. The synergistic effect was observed for almost all isolates by time-kill assay, except for combinations with tigecycline, whose synergistic effect was observed with tigecycline and colistin combinations only for susceptible-colistin isolates. The tigecycline with amikacin combination occurred against 17/20 isolates, and tigecycline with meropenem against ten isolates. For fosfomycin combinations, the synergistic effect was not observed when tested with vancomycin and with meropenem. Fig 1 shows the time-kill curve results.

For all isolates there was a synergistic effect of antibiotic combinations at 2 x MIC and 4 x MIC, except for one isolate using tigecycline-gentamicin and one isolate using colistin-gentamicin. For combinations at 0.125 x MIC and 0.25 x MIC, the synergistic effect was observed only for colistin-vancomycin and colistin-rifampicin.

A wide diversity of OXA-genes (OXA-51, OXA-23, OXA-72, OXA-88, and OXA-182) was found by whole genome sequencing as well as aminoglycoside resistance genes and genes encoding a series of proteins for the resistance-nodulation-division (RND) family and their regulators (Table 3). This Whole Genome Shotgun project was deposited at the DDBJ/EMBL/GenBank under the accession LMZH00000000 (Ab03), LMBM00000000 (Ab06), LMBN00000000 (Ab18), LMBO00000000 (Ab23), LMBP00000000 (Ab25), and LMBQ00000000 (Ab28). The carbapenem-resistant isolate with the highest MIC for carbapenem co-harbored OXA types and IMP. On the other hand, less diversity of OXA genes was found among colistin-resistant isolates.

Clinical, demographic, and treatment data of 18 patients were evaluated. Of these, three patients were colonized, and one died before the culture result; thus, a total of 14 patients received antibiotic therapy. Eight patients died during treatment and five during hospitalization; therefore, five continued in the investigation. The most frequent site of infection was the blood, in 13/14 patients. Seven patients received vancomycin plus specific therapy (colistin and or ampicillin/sulbactam) against *A*. *baumannii*; however mortality did not differ for this group compared with the group that did not receive vancomycin, despite the high *in vitro* synergism of colistin-vancomycin (Table 6).

**Table 6:** Clinical, demographic, and vancomycin time-kill synergism data of 18 patients colonized and infected by multidrug-resistant *Acinetobacter baumannii*.

	Patients N = 18	Surviving during treatment N = 6	Death during treatment N = 8
Age (years), mean (range)	43 (15–68)	38 (16–59)	44 (15–69)
Female	6	5	1
**Underlying diseases**			
**Hematologic cancer**	4	0	4
Cirrhosis	3	3	0
Abdominal surgery	2	1	1
Trauma	2	2	0
Kidney transplant	1	1	0
Others	5	2	3
Intensive Care Unit	6	4	2
Apache II, median	16	15	13
Colonization	3	-	-
No treatment	4*	-	-
Infection	14	6	8
Blood	13	6	7
Tracheal secretion	1	-	1
Treated *A*. *baumannii* infection	14	6	8
*In vitro* synergism with vancomycin plus colistin by time-kill	14	6	8
*In vitro* synergism with vancomycin plus imipenem by time-kill	9	4	5
Received vancomycin plus *A*. *baumannii* treatment	7	2	5

*Three colonization (one blood specimen collected by central venous catheter and two fecal specimens) and one patient with a bloodstream infection that died before treatment.

## Discussion

The combinations of colistin plus rifampicin, and colistin plus vancomycin showed the highest synergistic effect against colistin-resistant *A*. *baumannii* isolates. The synergistic effect *in vitro* by time-kill analysis occurred at a concentrations lower (0.5 and 1.0 x of the MIC values) than those used in clinical treatment (2.0 to 8.0 x the MIC values). Mortality among patients during treatment of infections due to MDRAB was high, despite high *in vitro* synergism with vancomycin, considering the use of vancomycin plus an active drug against *A*. *baumannii*.

Different interpretation procedures can be used to determine the effect of the antimicrobial combinations, but these methods may lead to different results. Therefore, we chose FICI and 2-well, the most frequently used methods in literature, and the time-kill method, which is considered the gold-standard [26]. We found different results comparing 2-well and FICI as demonstrated by other authors [27]. Bonapace et al. [27] demonstrated a 72% (range 42–97%) agreement between the time-kill test and Etest, and 51% (range 30–67%) between the time-kill and checkerboard tests. Thus, it seems that for clinical purposes, it may be important to confirm checkerboard results with time-kill testing.

To the best of our knowledge, this is the first study that evaluated the synergistic effect of fosfomycin using the time-kill assay against well-characterized colistin-susceptible and resistant *A*. *baumannii* isolates. We found a synergistic effect with fosfomycin and aminoglycoside against colistin-susceptible and resistant *Acinetobacter* isolates. However, we could not evaluate the use of fosfomycin in our patients because it is not yet approved to be used as systemic treatment in Brazil.

A recent study showed that patients who received combination therapy with colistin and fosfomycin had a significantly more favorable microbiological response and a trend towards more favorable clinical outcomes and lower mortality than those who received colistin alone [28–29]. Nevertheless, the use of fosfomycin to treat infections due to *Acinetobacter* is controversial. Data using intravenous fosfomycin are scarce, *Acinetobacter* usually shows high MICs, and breakpoints are not well defined by CLSI [10] or EUCAST [11]. Few studies have evaluated the synergistic effect of fosfomycin combined with colistin against *A*. *baumannii* [30–31], which may vary from 37.5% to 75% [30–31]. Despite all limitations, our data demonstrated a high rate of synergy of fosfomycin-aminoglycoside combinations against colistin-susceptible and resistant *Acinetobacter* isolates. This suggests that these combinations may be clinically useful.

In the present study, all colistin-resistant *A*. *baumannii* isolates harboring *bla*_OXA-23-like_ belonged to CC113 (ST233). This differed from prior reports that identified colistin-resistance in ST2 of the international clone II [32] and ST375 [33] in Italy and in Korea [32–33]. On the other hand, the colistin-susceptible *A*. *baumannii* isolates in our study that harbored *bla*_OXA-23-like_ belonged to CC103 (ST236). This CC has already been reported by Coelho-Souza et al. [34] in 2003 in another state of Brazil. Most OXA-23- producing *A*. *baumannii* isolates in Latin America belong to CC113, in contrast with studies in many countries around the world that showed that *A*. *baumannii* harboring OXA-23 isolates were related to specific clones belonging to CC92 [35–42].

OXA-23 and OXA-143 were the most frequent resistance mechanisms found in our isolates. *A*. *baumannii* harboring OXA-23 has been reported in many countries including Brazil, and it is associated with resistance to imipenem [43–47]. This is the first report of OXA-88 and OXA-182 among clinical isolates of *A*. *baumannii* in Brazil. OXA-88 sequences differ from OXA-51 by five to eight amino acids. It has been described among clinical isolates in Singapore [48]. OXA-182 has been reported among *A*. *baumannii* isolates in Korea and revealed 93% nucleotide identity with *bla*_OXA-143_ [49]. Other carbapenemase found in our isolates was OXA-72, already described in Brazil [50–52].

Outer-membrane proteins of 43 kDa previously associated with carbapenem resistance in *Acinetobacter* spp. [53] were absent in four isolates analyzed in the present study. These isolates showed a high MIC for imipenem and an indifferent effect of combinations with carbapenem. However, in all isolates, fosfomycin-amikacin and tigecycline-amikacin combinations were synergistic.

In our study, the combination of colistin with rifampicin showed the highest synergistic effect against colistin-resistant *A*. *baumannii*. This combination has already been suggested for treatment of MDRAB by both *in vitro* and *in vivo* studies, mainly in case series [54]. There is only one prospective small randomized trial that showed a higher rate of microbiological cure in the colistin and rifampicin combination group (71% of 15 patients) compared to the colistin group (59% of 13 patients) [55]. However, due to the high incidence of tuberculosis infection in our country, the use rifampicin is restricted and not used to treat *A*. *baumannii* infections.

In this study, we detected a synergistic effect of colistin associated with vancomycin in 80% of isolates by using the 2-well method. These results were confirmed by the time-kill assay, considered the gold standard. Three previous studies reported the activity of a combination of colistin with vancomycin against MDRAB; however, they analyzed a very small number of *A*. *baumannii* isolates [56–58]. One study evaluated six *A*. *baumannii* isolates by the checkerboard method [56], and synergism was detected in four. The other study included only three colistin-resistant *A*. *baumannii* isolates and showed a synergistic effect for all [58]. A study conducted by Vidaillac et al. [57] showed that all tested combinations including colistin-vancomycin were synergistic against four isolates of *A*. *baumannii*. According to these authors, colistin would disrupt the outer membrane and could facilitate glycopeptide penetration across the outer membrane, thus exposing the target site in the cell wall. The synergistic effect of colistin and vancomycin may be clinically useful in the intensive care setting because the empiric combination for septic patients usually includes a beta-lactam plus vancomycin. In hospitals with high MDR rates, polymyxin is added. On the other hand, this combination may involve risks such as acute kidney damage, and its impact on mortality has not yet been clearly demonstrated [58]. Although we found a high *in vitro* rate of synergism with vancomycin, death among patients who received vancomycin did not differ from that of those who did not receive it. These results could be due to the small number of patients evaluated, as well as the site of infection. The types of resistance mechanism among *A*. *baumannii* isolates could explain, at least in part, why combination regimens reported as successful in literature were not successful in our assays.

In our study, although we found a high rate of susceptibility to tigecycline (only 5% of isolates were resistant to this antibiotic), few antibiotic combinations using tigecycline showed a synergistic effect. The highest synergistic effect achieved with tigecycline was in combination with amikacin, similar to a prior study reported by Petersen et al. [9]. Thus, it would seem that tigecycline is not a good option to use in a combination to treat infection due to *A*. *baumannii* harboring OXA-23 and OXA-143, the most frequent resistance mechanisms identified in our isolates.

Our study has several limitations, such as the retrospective design of the study and the number of patients evaluated. We tried to evaluate the impact of “*in vitro* synergism” of vancomycin on death; however, we only had information on 18 patients, and only seven received vancomycin plus specific treatment against *A*. *baumannii*. The colistin-resistant isolates were closely related, the colistin-susceptible isolates belonged to different clones, but the synergistic effect was similar for isolates showing the same resistance mechanism.

In conclusion, our study demonstrates that colistin plus rifampicin, and colistin plus vancomycin showed the highest synergistic effect against colistin-resistant *A*. *baumannii* isolates. Among colistin-susceptible isolates, the highest synergistic effect was achieved with fosfomycin combined with amikacin or with gentamycin, and colistin combined with rifampicin or with vancomycin. Moreover, the synergistic effect against MDRAB appears to be related to the resistance mechanism and not to the clonality of isolates. Despite the high synergism *in vitro* with vancomycin, mortality of patients did not differ when comparing the use or not of vancomycin plus an active drug against *A*. *baumannii*.

## Supporting Information

S1 Table Antimicrobials susceptibility established by CLSI and EUCAST. (DOCX)
