# Supplementary material for: Antimicrobial Combinations against Pan-Resistant Acinetobacter baumannii Isolates with Different Resistance Mechanisms
Source: PLoS One. 2016 Mar 21;11(3):e0151270. doi: 10.1371/journal.pone.0151270 (PMC4801211; doi:10.1371/journal.pone.0151270)
Supplement: S1 Table — (DOCX) [file pone.0151270.s001.docx]

| ATB | Susceptibility (mg/L) | | |
| --- | --- | --- | --- |
|  | **Resistance** | **Intermediate** | **Sensibility** |
| Colistin | ≥4 | - | ≤2 |
| Imipenem | ≥16 | 8 | ≤4 |
| Meropenem | ≥16 | 8 | ≤4 |
| Tigecycline | ≥8 | 4 | ≤2 |
| Gentamycin | ≥16 | 8 | ≤4 |
| Amikacin | ≥64 | 32 | ≤16 |
| Rifampicin^b^ | ≥4 | 2 | ≤1 |
| Vancomycin^a^ | ≥16 | 4 - 8 | ≤2 |
| Fosfomycin^b^ | >32 | - | <32 |

^a^Criteria established by CLSI, 2013 for *Staphylococcus aureus,* ^b^Criteria established by EUCAST, 2013 for *Enterobacteriaceae.*
